# Glycerol content within the WHO ethanol-based handrub formulation: balancing tolerability with antimicrobial efficacy

**DOI:** 10.1186/s13756-019-0553-z

**Published:** 2019-06-24

**Authors:** Mayra Gonçalves Menegueti, Ana Maria Laus, Márcia Aparecida Ciol, Maria Auxiliadora-Martins, Anibal Basile-Filho, Elucir Gir, Daniela Pires, Didier Pittet, Fernando Bellissimo-Rodrigues

**Affiliations:** 1 Infection Control Service, University Hospital of Ribeirão Preto Medical School, University of São Paulo, Ribeirão Preto, Brazil; 2 Ribeirão Preto Nursing School, University of São Paul, Ribeirão Preto, Brazil; 3 University of Washington, Seattle, USA; 4 Intensive Care Division, Ribeirão Preto Medical School, University of São Paulo, Ribeirão Preto, Brazil; 5 Infection Control Programme, University of Geneva Hospitals and Faculty of Medicine, Geneva, Switzerland; 6 Social Medicine Department, Ribeirão Preto Medical School, University of São Paulo, Ribeirão Preto, Brazil; 7 Escola de Enfermagem de Ribeirão Preto, Universidade de São Paulo, Campus Universitário, s/n, Monte Alegre, 14048-900, Ribeirão Preto, São Paulo, Brazil

**Keywords:** Hand hygiene, World Health Organization, Alcohol-based hand rub, Glycerol, Skin tolerability, Essential medicines list, Low-income countries

## Abstract

**Background:**

The World Health Organization (WHO) ethanol-based handrub (EBHR) formulation contains 1.45% glycerol as an emollient to protect healthcare workers’ (HCWs) skin against dryness and dermatitis. However, glycerol seems to negatively affect the antimicrobial efficacy of alcohols. In addition, the minimal concentration of glycerol required to protect hands remain unknown. We aim to evaluate the tolerance of HCWs to the WHO EBHR formulation using different concentrations of glycerol in a tropical climate healthcare setting.

**Methods:**

We conducted a cluster-randomized, double-blind, crossover study among 40 HCWs from an intensive care unit of a tertiary-care hospital in Brazil*,* from June 1st to September 30, 2017. We tested the WHO EBHR original formulation containing 1.45% glycerol against three other concentrations (0, 0.5, and 0.75%). HCWs used one formulation at a time for seven working days during their routine practice and then had their hands evaluated by an external observer using the WHO scale for visual inspection. Participants also used a WHO self-evaluation tool to rate their own skin condition. We used a generalized estimating equations of the logit type to compare differences between the tolerability to different formulations.

**Results:**

According to the independent observation, participants had 2.4 times (95%CI: 1.12–5.15) more chance of having a skin condition considered good when they used the 0.5% compared to the 1.45% glycerol formulation. For the self-evaluation scale, participants were likely to have a worst evaluation (OR: 0.23, 95%CI: 0.11–0.49) when they used the preparation without glycerol compared to the WHO standard formulation (1.45%), and there were no differences between the other formulations used.

**Conclusion:**

In a tropical climate setting, the WHO-modified EBHR formulation containing 0.5% glycerol led to better ratings of skin tolerance than the original formulation, and, therefore, may offer the best balance between skin tolerance and antimicrobial efficacy.

## Background

Alcohol-based handrub (ABHR) is universally available in high-income countries, where many formulations with different type and concentrations of emollients, different applications times and costs are easily found. However, the availability of these products in healthcare facilities from low- and middle-income countries is inconsistent. In several countries in Africa, Asia, and Latin America such products are unavailable or inaccessible due to their high cost [1].

In response to this issue, the World Health Organization (WHO) has developed two formulations that can be locally prepared by healthcare facilities. One formulation contains ethanol 80% (v/v), glycerol 1.45% (v/v) and hydrogen peroxide 0.125% (v/v), and the other isopropanol 75% (v/v), glycerol 1.45% (v/v) and hydrogen peroxide 0.125% (v/v). In both preparations, the addition of glycerol as an emollient aims to protect the hand skin against dryness and dermatitis potentially resulting from repeated use [1]. This is very important because the occurrence of dermatitis in healthcare workers (HCWs) hands severely compromises compliance with hand hygiene procedures [2–4].

Importantly, however, the 1.45% glycerol content within both WHO ABHR formulations has been shown to reduce the antimicrobial efficacy of the alcohols in laboratory-based microbiological investigations [5, 6]. Furthermore, the minimal concentration of glycerol required to protect HCWs hands remains unknown and that minimum may vary according to the climate in which professionals are practicing.

We evaluated the skin tolerability of HCWs to the WHO ethanol-based handrub formulation (EBHR) using different concentrations of glycerol in a tropical climate healthcare setting.

## Methods

We conducted a cluster-randomized, double-blind, crossover study in a 9-bed general intensive care unit (ICU) of a tertiary-care hospital in Brazil, from June 1st to September 30, 2017.

All 45 HCWs (physicians, nurses, technicians and nursing assistants, and physiotherapists) who worked regularly in the ICU for a minimum of 20 h per week were invited and agreed to participate in the study. All study participants signed the informed consent form. The study was approved by the Research Ethics Committee of the School of Nursing of the University of São Paulo at Ribeirão Preto, according to Resolution 466/12 of the National Health Council (number: 64803917.6.0000.5393).

For feasibility reasons, all participants were exposed to the same EBHR formulation in each study period. The order of use of the products was randomized. Both the HCW and the rater were blinded to the concentration used in each study phase. The external rater was an infection control nurse with a large experience in hand hygiene promotion and observation, and specially trained for performing the study tasks attributed to her. During each phase, participants had access to the EBHR formulation being tested only, that was made available at the point of care, attached to the beds, and in the nursing stations, for 30 consecutive days. Participants still had the option of washing their hands with soap and water, if necessary. During all study phases, powder-free gloves were provided to HCWs so that they could use the available EBHR even after the use of gloves. Participants had their hands evaluated just after using each formulation for 7 working days during the study periods. This was the “per protocol” population.

**Fig. 1:**
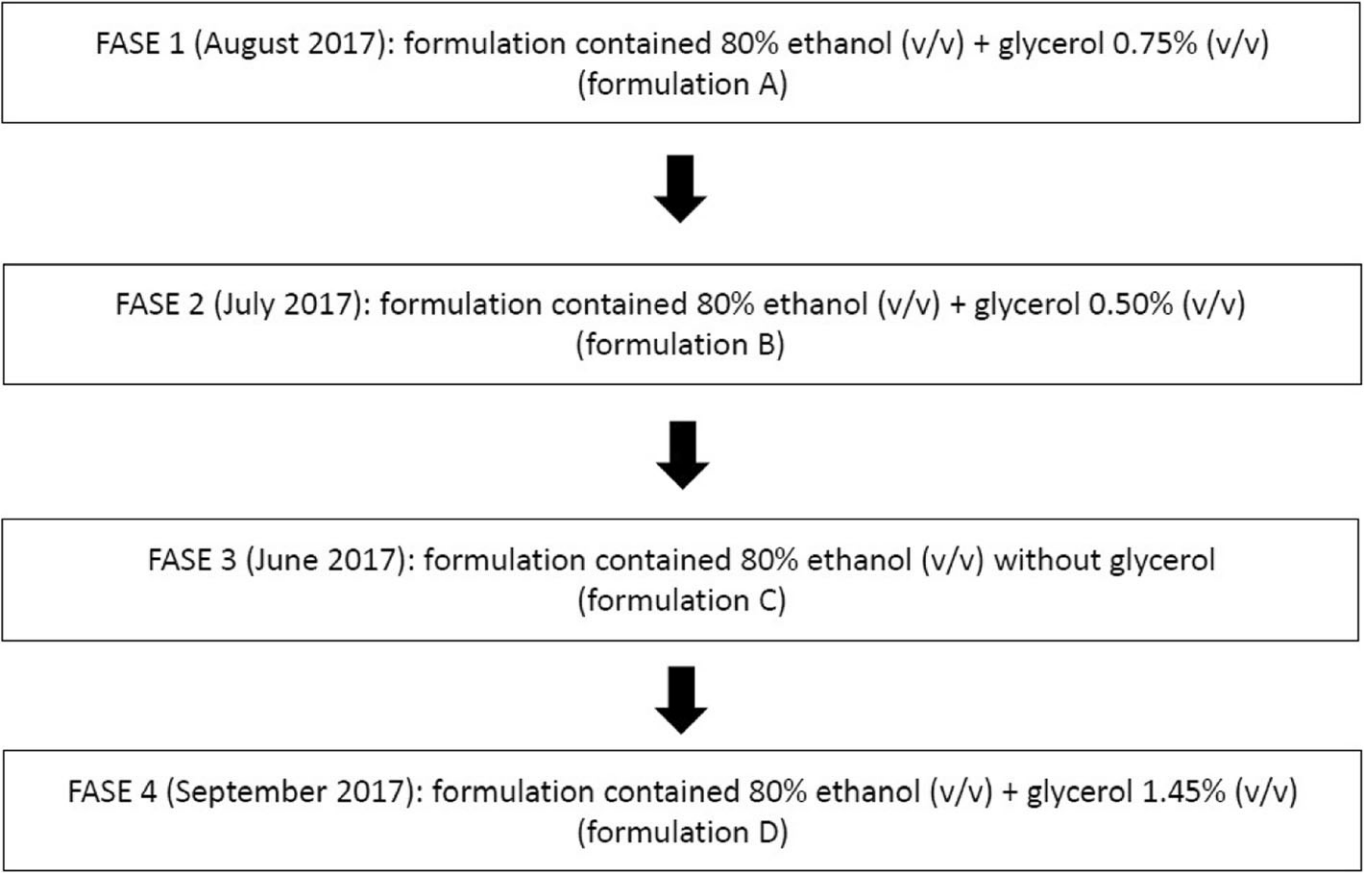
Phases of the study implementation

The formulations were tested sequentially, according to the order of randomization provided by the pharmacy department of the study hospital. All formulations contained 80% ethanol (v/v), with the addition of glycerol in various concentrations (v/v): 0% (denominated formulation C), 0.50% (formulation B), 0.75% (formulation A), and 1.45% (formulation D) [1]. The Fig. 1 illustrates the four phases of the study implementation. However, H_2_0_2_ was not used as a preservative due to the unpleasant odor it produced and considering that the EBHR bottles were subjected to cleaning and thermal disinfection prior to their reuse.

### Data collection instruments

All data collection instruments used for the purpose of the study were validated tools [7]. Questionnaires about demographic data and self-evaluation of the skin condition were completed by the participant her/himself. The HCW skin tolerance assessment form was completed by a single external rater. All skin assessments were performed after 7 days of use of each formulation. The two skin condition evaluation instruments contain items that are evaluated separately, with no total score per instrument.

For the characterization of the professionals and factors that could possibly interfere with skin condition, the questionnaire included the following items: sex, age, professional category, working time (full-time or part-time), skin type (Caucasian vs. not Caucasian), performing other activities that may cause skin damage, like gardening and bricolage with cement use (yes or no), use of protective hand lotion and/or cream at least once daily (yes or no), history of irritative dermatitis (yes or no), history of atopic dermatitis (yes or no), history of rhinitis and/or allergic conjunctivitis (yes or no), asthma (yes or no), and history of ABHR intolerance (yes or no) [7].

Assessment of skin tolerance (by external rater): this instrument is composed of four items: redness, ranging from 0 (not red) to 4 (very vivid red with edema); scaliness, ranging from 0 (not squamous) to 3 (very pronounced separation of the edges of the skin scale); fissures ranging from 0 (no cracks) to 3 (extensive cracks with bleeding or secretion, and visual score of skin); visual rating of skin from 0 (no irritation of any kind) to 5 (extensive cracking of the surface with bleeding and/or secretion) [7].

Self-assessment of skin condition (by participant): this instrument is composed by five items: appearance; integrity; humidity, sensation (all ranging from 1 [abnormal] to 7 [normal]), and general integrity (ranging from 1 [very compromised] to 7 [perfect]) [7].

Hand hygiene opportunities and compliance was measured by direct observation, as recommended by WHO, during each phase of the study implementation [8].

### Data analysis

Descriptive analyzes were made for each of the observed variables. The items observed by the rater were dichotomized in “without/with redness”, “without/with fissures”, “without/with scaliness” and “without/with visual rating of skin” according to the values observed (“without” if the score was 0 and “with” if the score was greater than or equal to 1). A dichotomous variable was created for rater evaluation: good tolerability (if all items above zero) or not good tolerability (at least one item with a score of 1 to 4).

A dichotomous variable was created for the self-evaluation of the skin condition: good condition (all items above receiving scores 6 or 7), and not good condition (at least one item receiving score 1 to 5).

The two dichotomous variables were analyzed separately as response variables using generalized estimating equations (GEE), with logit link, and unstructured covariance matrix. Glycerol concentration was included in the model as the explanatory variable and the results are presented as odds ratios for good outcome of any glycerol concentration compared to the WHO original formulation, containing 1.45% glycerol. The analysis considered the data as non-independent due to the intra-person correlation. All analyses were performed in STATA SE, version 14, and graphs were built using R Studio.

## Results

All the 45 potentially eligible participants were randomized and included in the study. However, five among them took a vacation period during the study implementation and could not complete 7 working days using one of the study formulations. Therefore, they were not included in the “per protocol” population, finally consisting in 40 HCWs. It is important to mention that no participant dropped out of the study; there was no severe adverse event related to the use of the study formulations.

The demographic data of the 40 HCWs who participated in all phases are shown in Table 1. Most of the participants were female (70%), and most were nurse assistants (70%).

**Table 1:** Selected baseline clinical and demographical characteristics of the healthcare workers participating in the study

Demographic and clinical characteristics (*n* = 40)	
Age, years, mean (standard deviation [SD])	39 (1.45)
Sex, female, n (%)	28 (70.0)
Profession, n (%)	
Medical doctor	4 (10.0)
Registered nurse	5 (12.5)
Auxiliary nurse	28 (70.0)
Physiotherapist	3 (7.5)
Working time, n (%)	
Full-time	18 (45.0)
Part-time (50–90%)	22 (55.0)
Work shift	
Day shift	26 (65.0)
Night shift	14 (35.0)
Skin type	
Caucasian	31 (77.5)
Non-Caucasian	9 (22.5)
Irritative dermatitis, n (%)	7 (17.5)
Atopic dermatitis, n (%)	1 (2.5)
Rhinitis and/or allergic conjunctivitis, n (%)	18 (45.0)
Asthma, n (%)	0
Intolerance to ABHR, n (%)	0
Non-work-related activities likely to affect the skin, n (%)	2 (5.0)
Regular use of protective hand lotion and/or cream, n (%)	23 (70.0)

*ABHR* alcohol-based handrub

The total number of observed opportunities and average compliance with WHO 5 moments for hand hygiene in each study phase were as follows: 1139 and 60.1 (SD [standard deviation]: 10.1%) for the formulation without glycerol; 1161 and 58.6% (SD: 10.2%) for the 0.75% glycerol formulation; 1200 and 67.6% (SD: 7.8%) for the 1.45% glycerol formulation; and 1138 and 60.6% (SD: 9.25%) for the 0.5% glycerol formulation, respectively.

**Fig. 2:**
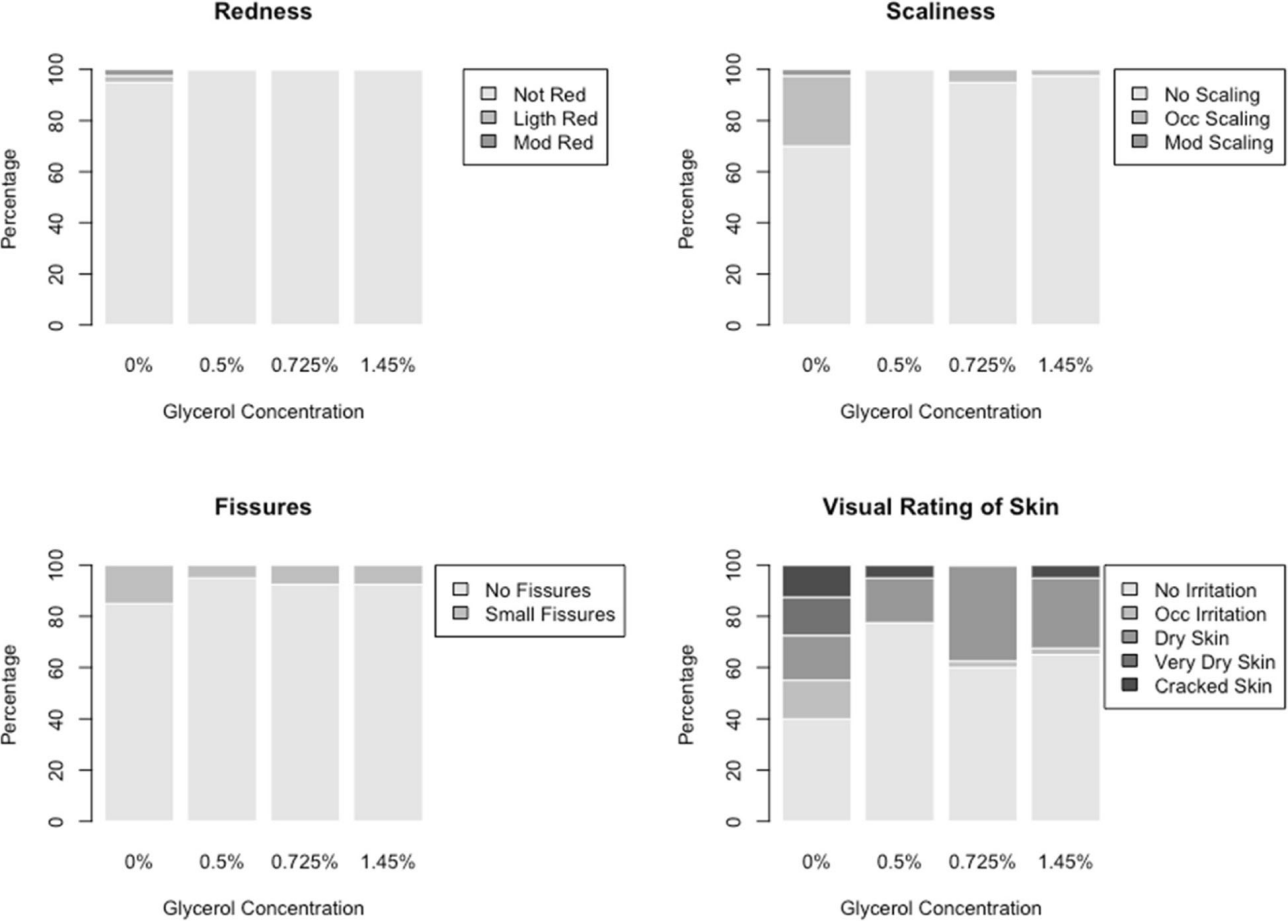
External rater evaluation of redness, scaliness, fissures, and visual rating of the skin by glycerol concentration; distribution of scoring results for 40 participants

The observer scores are shown in Fig. 2. Most of the skin reactions occurred in the non-glycerol formulation and included various reactions from occasional irritation to cracked skin. Table 2 shows the results for the dichotomous evaluations, as percentages of participants whose evaluation was considered “good”, by glycerol concentration. There was little variation in redness, scaliness, and fissures according to the glycerol concentration. The largest variation in ratings occurred for the visual rating of the skin, with worse results for the formulation without glycerol (37.5%), followed by 0.75% glycerol (62.5%), 1.45% glycerol (67.5%), and 0.5% glycerol (75%).

**Table 2:** Skin assessment by external rater and study participants across different glycerol concentrations in WHO ethanol-based handrub formulation

Ethanol-based handrub formulation	Without glycerol N^a^ (%)	Glycerol 0.5% N^a^ (%)	Glycerol 0.75% N^a^ (%)	Glycerol 1.45% N^a^ (%)
External rater evaluation				
No Redness	38 (95.0)	40 (100.0)	40 (100.0)	40 (100.0)
No Scaling	28 (70.0)	40 (100.0)	38 (95.0)	39 (97.5)
No Fissure	33 (82.5)	37 (92.5)	38 (95.0)	38 (95.0)
Visual rating of the skin	15 (37.5)	30 (75.0)	25 (62.5)	27 (67.5)
Self-evaluation				
Good appearance	15 (37.5)	32 (80.0)	26 (65.0)	30 (75.0)
Good integrity	34 (85.0)	37 (92.5)	38 (95.0)	37 (92.5)
Good humidity	17 (42.5)	30 (75.0)	29 (72.5)	30 (75.0)
Good sensation	35 (87.5)	38 (95.0)	39 (97.5)	38 (95.0)
Good general integrity	23 (57.5)	32 (80.0)	32 (80.0)	32 (80.0)

Footnote to the Table 2

^a^Express the number (N) and percentage of evaluation considered good for the assessment of the skin by the rater (rater evaluation) and the participants (self-evaluation of condition) according to the concentration of glycerol in the alcohol-based handrub formulation

**Fig. 3:**
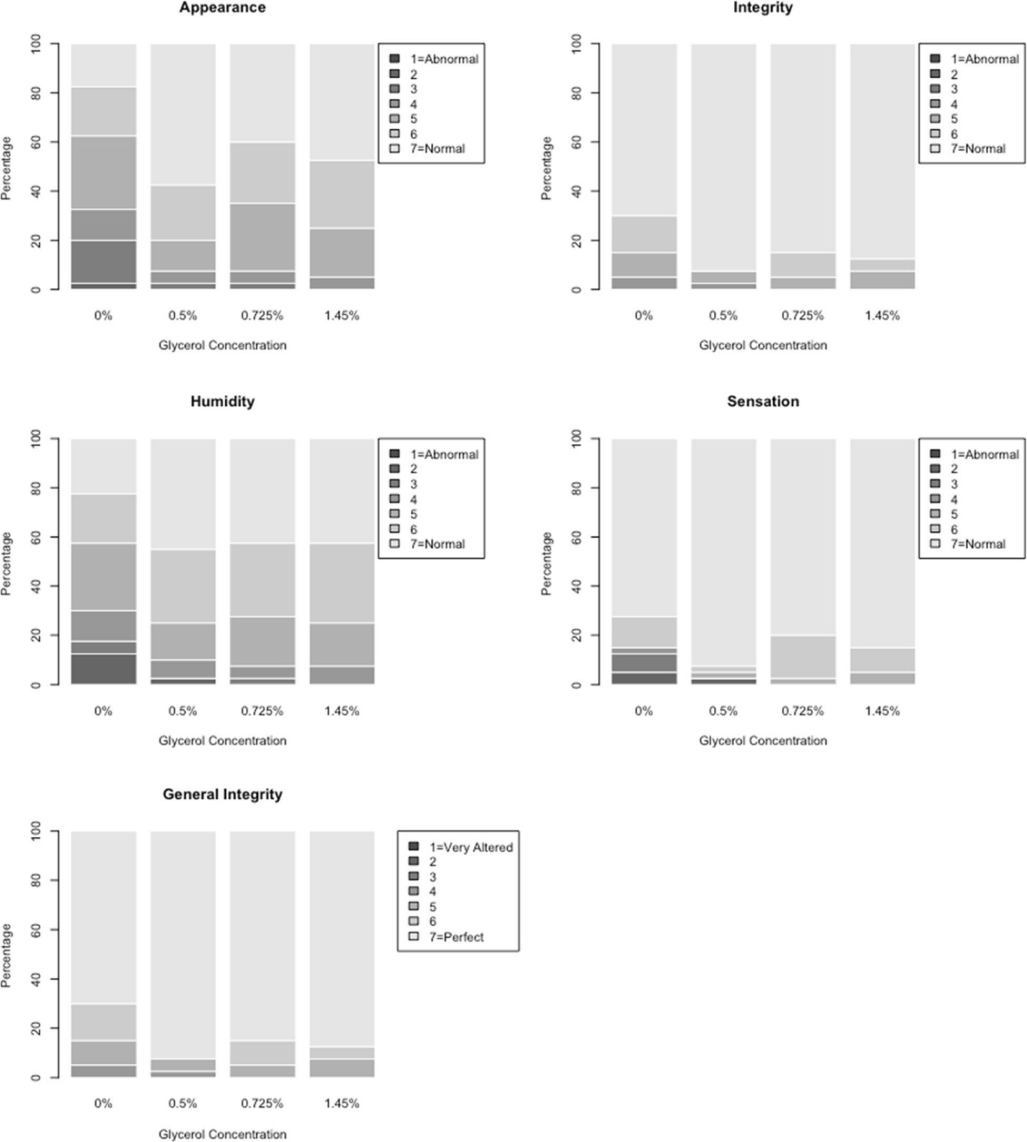
Self-evaluation of appearance, integrity, humidity, sensation, and general integrity of skin, by glycerol concentration; distribution of scoring results for 40 participants

Self-evaluation scores are shown in Fig. 3. Table 2 also shows the results for the self-evaluation items, as the percentage of participants endorsing good condition for each item, by glycerol concentration. For appearance, only 37.5% reported good condition when using a formulation without glycerol, while a minimum of 65% reported good condition for the other formulations. Figure 3 shows bar graphs with the percentage of each specific response within each EBHR formulation for self-evaluation (lighter colors correspond to better outcomes).

Appearance, humidity, and sensation were the items that had a few individuals with the worst ratings for the formulation without glycerol. Overall, the best formulation (the one with higher percentages of self-evaluation of good condition) was the formulation with 0.5% glycerol concentration.

Table 3 shows the results for the analyses of the two dichotomous variables defined as “good tolerability” for the external rater evaluation and “good condition” for the self-evaluation. In the rater evaluation, participants were likely to have a worse evaluation when using the solution without the emollient agent compared to the WHO standard solution (1.45%). In contrast, the participants had 2.4 times more chance of having a good evaluation when they used the 0.5% glycerol formulation compared to the 1.45% formulation.

**Table 3:** Model of generalized logistic estimation equations for evaluation considered good for the variables of the skin observation scale performed by the external rater and the study participant for the different alcohol-based formulations

Glycerol Concentration	Proportion with a good outcome	Model Coefficients	Odds Ratio (95% CI)^a^
External rater evaluation^b^			
Glycerol 0%	0.4	−0.92	0.20–0.79
Glycerol 0.5%	2.4	0.88	1.12–5.15
Glycerol 0.75%	0.9	−0.10	0.46–1.77
Glycerol 1.45%	1	–	–
Self-assessment^c^			
Glycerol 0%	0.23	−1.47	0.11–0.49
Glycerol 0.5%	1.12	0.11	0.53–2.36
Glycerol 0.75%	0.73	−0.32	0.35–1.51
Glycerol 1.45%	1	–	–

^a^95% CI = 95% confidence interval

^b^External rater evaluation: defined as “good” when all four ratings from rater evaluation were 0 (no redness, no scaling, no fissures, and no irritation)

^c^Self-assessment: defined as “good” when all five ratings from self-evaluation were 6 or 7 (normal or perfect)

In the self-assessment of the skin, participants were less likely to have a good evaluation when they used the preparation without the emollient compared to the WHO standard solution (1.45%), and there was no difference between the other formulations used.

## Discussion

Although hand hygiene is considered the cornerstone of infection prevention and control activities in healthcare settings, compliance with practices remains suboptimal worldwide [9–14]. One of the main barriers to hand hygiene promotion in low- and middle-income countries is the inconsistent availability of good quality ABHR, at affordable prices [1].

To overcome this barrier and assure universal accessibility, WHO has added ethanol to the Essential Medicine’s List [15] and developed two formulations of ABHR to be locally prepared by healthcare facilities. In both preparations, the addition of glycerol as an emollient aims to protect the hand skin against dryness and dermatitis sometimes associated with repeated use [1]. Skin damage in HCWs hands is a serious problem, placing both the professional and the patient at higher risk of acquiring healthcare-associated infections and colonization with multidrug resistant pathogens. In view of the relevance of this problem, the United States public health service aims to reduce the incidence of skin lesions in HCWs hands by 10 % by 2020, with the reference rate being 4.4 per 10,000 full-time workers in 2008 [16].

A previous study evaluating the tolerability and acceptability of the WHO ABHR formulations proved them to be very well tolerated by HCWs [17]. Importantly, however, the 1.45% glycerol content within both WHO ABHR formulations has been shown to significantly reduce the antimicrobial efficacy of the alcohols in laboratory-based microbiological investigations [5, 6]. To the best of our knowledge, it is not exactly known what is the cause of this interaction but possible explanations have been proposed. Among them, glycerol could react with flaking skin cells forming sticky agglomerates that may protect bacteria from being exposed to the alcohol. This phenomenon has not been observed in the current study. Another possible explanation is that a large part of the normal skin microbiota is able to ferment glycerol, and, therefore, glycerol could act as a growth factor for the residual bacteria surviving the alcohol exposure [6].

Thus, it is important to know what would be the minimal concentration of glycerol required to protect HCW hands from dryness and dermatitis. Such minimum may vary according to the climate in which the professional is working. In cold-climate settings, the HCWs skin tends to be drier than in tropical areas. Beyond that, the use of hot tap water for washing hands, which is frequent in many cold countries, may turn skin even drier. In this regards, it is relatively common for HCWs in our hospital in Brazil to complain of a “sticking” effect when using ABHRs developed for cold-climate countries with high concentrations of emollient agents (unpublished data). A study identified that the degree of hand dryness in the fingers’ joints and in the hands dorsum was significantly higher in the winter than in the spring in particular, skin erythema decreased during spring and increased during winter [18].

Considering that most of the low- and middle-income countries are located in tropical and subtropical areas of Africa, Asia and Latin America, where locally-produced WHO ABHR formulations are needed the most, we decided to conduct the current study in a tropical climate healthcare setting. Our results clearly indicate that the addition of glycerol to the WHO formulation is important for the maintenance of the HCWs skin integrity, as shown previously by others [19]. The formulation without glycerol yielded significantly worse skin tolerance scores than the original formulation, by both independent evaluation and self-assessment. According to our results, the 0.5% glycerol was the minimal concentration studied yielding scores of skin tolerance equivalent or better than the original formulation.

The current study has major strengths. First, we used validated tools for assessing the HCWs skin condition, as recommended in the WHO Guidelines for Hand Hygiene in the Healthcare Setting [8, 17, 20, 21]. In previous studies, these tools exhibited an excellent correlation with physiological measures of skin evaluation, such as skin water loss and transepidermal evaluation [2, 21, 22]. Furthermore, we conducted the study in real-life conditions during a sufficient period of time for cumulative skin adverse effects to happen (7 working days for each tested ABHR formulation). According to Larson and colleagues [23], 3 working days is the minimum period necessary to effectively compare the skin tolerance of different agents for hand hygiene, in high workload conditions with a high number of hand hygiene opportunities per hour of patient care [23].

The present study is subject to some limitations. It was performed in a single center with a sample of 40 HCWs. Therefore, larger studies are necessary to confirm these results. Another limitation was that we did not evaluate the volume of EBHR used, nor the friction time, that are important determinants of the quality of hand decontamination and may influence the occurrence of adverse events [24]. Finally, we cannot totally rule out the possibility of a carrying-over effect, i.e., the residual effect of one of the formulations affecting the results of the next one. To minimize this possibility, all participants were assessed only after having used the formulation for at least 7 working-days, believing it would be enough to dissipate the effects of the previous formulation.

Future studies should confirm that the 0.5% glycerol WHO-modified EBHR formulation is microbiologically superior to the WHO original formulation, as expected by extrapolation of the results from studies by Suchomel and colleagues [5, 6].

## Conclusion

In conclusion, our results demonstrate that, in a tropical climate setting, the WHO-modified EBHR formulation containing 0.5% glycerol led to better ratings of skin tolerance than the original formulation, and, therefore, may offer the best balance between skin tolerance and antimicrobial efficacy.

## Data Availability

The anonymised datasets analysed during the current study are available from the corresponding author (FBR; fbellissimo@fmrp.usp.br) on reasonable request, as long as this meets local ethics and research governance criteria. All data generated or analyzed during this study are included in this published article.

## Abbreviations

95%CI: 95% Confidence Interval
ABHR: Alcohol-based hand-rub
EBHR: Ethanol-based hand-rub
HCWs: Healthcare workers; v/v: volume/volume
ICU: Intensive care unit
N: Number
SD: Standard deviation
WHO: World Health Organization
